# Striving for scientific excellence in hand surgery

**DOI:** 10.1177/1753193420927581

**Published:** 2020-05-29

**Authors:** Florian S. Frueh, Jason K. Wong, Kai Megerle, Shai Luria, Simon Farnebo

**Affiliations:** 1 Department of Plastic Surgery and Hand Surgery, University Hospital Zürich, Zürich, Switzerland; 2 Blond McIndoe Laboratories and Department of Plastic Surgery, University of Manchester and Manchester University Foundation Trust, Manchester, UK; 3 Divison of Hand Surgery, Klinikum rechts der Isar, Technical University of Munich, Munich, Germany; 4 Department of Orthopedic Surgery, Hadassah-Hebrew University Medical Center, Jerusalem, Israel; 5 Department of Plastic Surgery, Hand Surgery, and Burns, Linköping University, Linköping, Sweden

The research committee of the Federation of European Societies for Surgery of the Hand (FESSH) recently reported the results of a survey on research activities of European hand surgeons (Frueh et al., 2019). The survey of more than 500 hand surgeons identified two common factors limiting the success of academic activity in hand surgery: the lack of dedicated research time (71%) and the lack of funding (48%). To stimulate and facilitate research efforts in hand surgery, the research committee of FESSH initiated a number of research grants, which it aims to award annually. For 2020, four applications were awarded grants totalling €70,000.

## Research grants currently available from FESSH

### Basic Research Grant

The basic research grant was initiated to encourage pump priming of exploratory, translational, or developmental research, supporting in particular early stages of new preclinical or clinical approaches. Hence, it is directed towards basic and translational science and novel ideas or techniques in hand surgery. Currently the awarded sum is €10,000 for a 1-year grant.

### Clinical Research Grant

This grant aims to support clinical research projects in all areas of hand surgery. Clinical projects that are directed towards outcomes or assessment of treatment modalities in topics related to hand surgery will be considered. Well-designed feasibility and prospective trials will be promoted. The awarded sum is also €10,000 for a 1-year grant.

### FESSH/Foundation for Hand Surgery Clinical Research Grant

This grant aims to support novel hand surgery research with a clear line of sight for patient benefit, with a comparable scope with the above-mentioned clinical research grant. The awarded sum is €50,000 for 1 year. If deemed appropriate by the committee, this sum may be divided into multiple 1-year grants. Furthermore, the Foundation has invited and encouraged funded projects to reapply directly to the Foundation for extended long-term support.

The Foundation for Hand Surgery (https://www.foundation-handsurgery.org) is a non-profit organization based in Geneva, Switzerland. Its mission is to develop and improve hand, wrist, and upper limb surgery. It devotes resources to develop research axes that bring new concepts and innovative medical and surgical solutions for hand and wrist surgery over the next decades. Moreover, the Foundation for Hand Surgery promotes hand surgery education by establishing an interactive European centre of excellence, including a unique curriculum, with hands-on courses for surgeons at all stages of their professional training. The vision is to develop centres dedicated to hand surgery in countries that presently have none.

## 2020 Grant Winners

This year the Basic Research Grant has been awarded to *Dr Ryan Trickett* and his team (Cardiff University, UK). Dr Trickett's project relates to kinematics at the base of the normal thumb: an analysis of healthy thumbs combining bi-planar fluoroscopy, motion capture, and three-dimensional (3-D) modelling will explore the kinematics of the healthy thumb base. The project aims at combining different modalities to create a 3-D reconstruction of the complex joint structure and its kinematics in various motions.

The Clinical Research Grant has been awarded to *Dr Thorsten Schriever* and his team (Department of Hand Surgery at Södersjukhuset, Stockholm, Sweden). Dr Schriever's research project is a randomized comparison between two common treatment options – lunocapitate arthrodesis versus four-corner arthrodesis – for scaphoid non-union advanced collapse and scapholunate advanced collapse arthritis.

One FESSH/Foundation for Hand Surgery Clinical Research Grant has been awarded to *Dr Olga Politikou* and her team (Clinical Laboratory for Bionic Extremity Reconstruction, Department of Surgery, Medical University of Vienna, Austria). They are studying nerve transfers for cognitive reinnervation of spastic muscles in stroke patients. A second FESSH/Foundation for Hand Surgery Clinical Research Grant has been awarded to *Dr Brigitte van der Heijden* and her team (Radboud University Medical Center, Nijmegen, The Netherlands). They are investigating the diagnostic performance of dynamic four-dimensional computed tomography compared with arthroscopy for analysing scapholunate instability.

The FESSH research committee strongly encourages the submission of basic and clinical research grants for the 2021 application period, and such grants will now be awarded annually. We are well aware that applying for dedicated research time and funding is exhausting, but this is a much-needed part of a clinician-scientist's professional activity. As reflected in the above listed awardees, the clinical grant application should aim to elucidate a key clinical question or explore a novel clinical intervention through carefully designed clinical studies and with sufficiently long follow-up if outcome measures are necessary. In our speciality there are many unanswered questions, high-quality clinical studies are much sought after, and long clinical follow-up is essential (Long et al., 2018; Tang, 2019; Tang et al., 2019). In the awardee lists, the clinical and basic science teams almost all include international participants. This provides hand surgeons with much sought international training opportunities, as encouraged by our senior colleagues (Garcia-Elias and Tang, 2018). We finally would like to express our deep gratitude to all colleagues who are continuously expanding the limits and enhancing excellence in hand surgery.
